# A Rare Case of Non-Hodgkin Lymphoma With Ankle Involvement: Diagnostic and Therapeutic Challenges

**DOI:** 10.7759/cureus.77987

**Published:** 2025-01-25

**Authors:** Arın Celayir, Muhammed Yusuf Afacan, Baran Sevgil, Mahmut K Ozsahin, Huseyin Botanlioglu

**Affiliations:** 1 Department of Orthopaedics and Traumatology, Istanbul University-Cerrahpasa, Cerrahpasa Faculty of Medicine, Istanbul, TUR

**Keywords:** ankle involvement, biopsy, chemotherapy, non-hodgkin lymphoma, radiotherapy

## Abstract

Non-Hodgkin lymphoma (NHL) is a diverse group of malignancies primarily originating in the lymphatic system. Musculoskeletal involvement, especially in joints such as the ankle, is exceedingly rare. Accurate diagnosis is often complicated by overlapping features with other conditions like osteomyelitis or malignancies. This case report aims to highlight the diagnostic and therapeutic challenges associated with joint-related NHL presentations.

A 49-year-old man presented with a three-month history of left ankle pain. Initial imaging, including contrast-enhanced magnetic resonance imaging (MRI), suggested osteomyelitis. To exclude malignancy, a whole-body positron emission tomography (PET) scan was performed, which indicated a high suspicion for malignancy. An open biopsy was conducted, and samples were analyzed for pathology and microbiology. The pathological evaluation revealed high-grade B-cell NHL. Preoperative laboratory findings were unremarkable, but further hematological workup confirmed the diagnosis. The patient was referred to oncology and commenced on a combination of chemotherapy and radiotherapy. At the six-month follow-up, the patient reported significant pain relief, and follow-up PET-computed tomography (CT) imaging showed no evidence of recurrence or metastasis.

Although rare, NHL should be considered in the differential diagnosis of joint and bone lesions. This case highlights the importance of a multidisciplinary approach, including radiological, pathological, and oncological evaluations, in ensuring accurate diagnosis and effective treatment planning. Early intervention and targeted therapy significantly improved the patient's outcome.

## Introduction

Non-Hodgkin lymphoma (NHL) encompasses a broad spectrum of cancers originating within the lymphatic system and primarily impacting lymphocytes, which are essential white blood cells involved in immune defense. Unlike Hodgkin lymphoma, NHL is characterized by the absence of Reed-Sternberg cells, a defining feature of Hodgkin lymphoma. However, immunohistochemistry (IHC) plays a crucial role in accurately differentiating between NHL and Hodgkin lymphoma by identifying specific cellular markers that provide definitive diagnostic clarity beyond the presence or absence of Reed-Sternberg cells [1]. NHL can develop from various lymphocyte types, including B cells, T cells, and natural killer cells, giving rise to numerous subtypes with unique clinical features and treatment requirements [2]. Clinical manifestations of NHL depend on the specific subtype and the disease's location but commonly include enlarged lymph nodes, fever, night sweats, fatigue, and unexplained weight loss. The diagnostic process typically incorporates imaging modalities, such as computed tomography (CT) and positron emission tomography (PET) scans, alongside histopathological analysis of biopsy specimens [3]. Treatment strategies for NHL are individualized and may involve chemotherapy, radiation therapy, immunotherapy, targeted therapy, or stem cell transplantation, depending on the patient's disease subtype, stage, and overall health condition [4].

NHL affecting the musculoskeletal system is relatively rare and poses significant diagnostic and therapeutic challenges. The prognosis depends on factors such as the NHL subtype, the extent of disease involvement, and the patient's response to treatment.

In this report, we detail the treatment administered to a patient who presented to our clinic with ankle pain and was subsequently diagnosed with NHL following biopsy surgery.

## Case presentation

**Figure 1:**
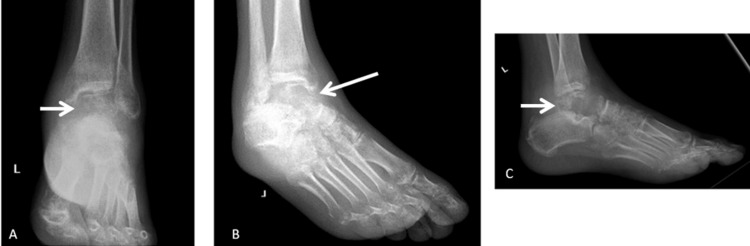
X-ray images of the patient at the time of hospital admission: (A) AP view of the ankle, (B) mortise view of the ankle, and (C) lateral view of the ankle. The white arrows indicate the area of the lesion. AP: anterior-posterior

A 49-year-old man presented with complaints of persistent pain in the left ankle lasting for three months. He reported no associated constitutional symptoms such as weight loss, fatigue, or night sweats. Additionally, there was no history of fever, prior infection, or a local wound in the affected area. Upon his initial visit, a thorough clinical evaluation was conducted, and three-view ankle radiographs were requested to assess the underlying cause of the symptoms. Informed consent was obtained from the patient before any diagnostic or therapeutic procedures were performed. Irregularities observed in the talus on the radiographs raised suspicion of possible malignancy or osteomyelitis, prompting a request for magnetic resonance imaging (MRI) to aid in differentiation (Figure 1).

**Figure 2:**
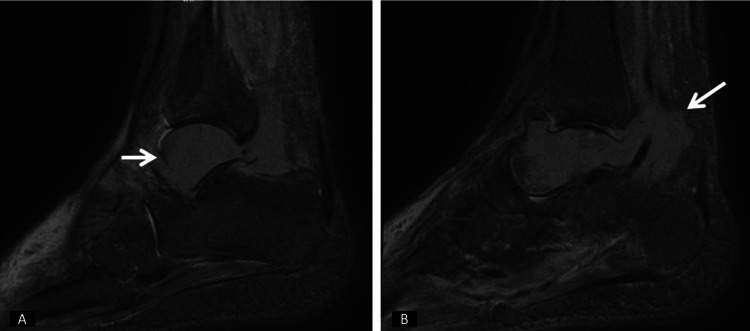
Sagittal MRI of the left ankle, obtained with T2-weighted sequences following contrast administration, shows abnormal signal intensity and contrast enhancement in the distal tibia, talus, and surrounding soft tissues, initially suggestive of osteomyelitis. (A) demonstrates involvement around the talus, while (B) highlights involvement in the soft tissue surrounding the talus. The white arrows indicate the area of the lesion. MRI: magnetic resonance imaging

**Figure 3:**
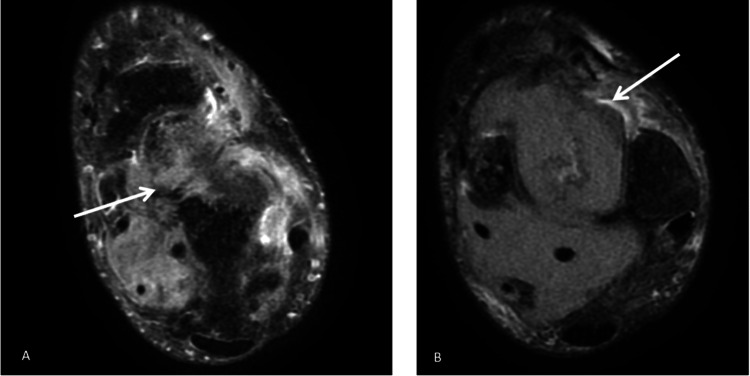
Axial MRI of the left ankle, obtained with T2-weighted sequences following contrast administration, reveals abnormal signal intensity and soft tissue involvement surrounding the distal tibia, consistent with an aggressive lesion. (A) demonstrates involvement around the talus, while (B) highlights involvement in the soft tissue surrounding the talus. The white arrows indicate the area of the lesion. MRI: magnetic resonance imaging

**Figure 4:**
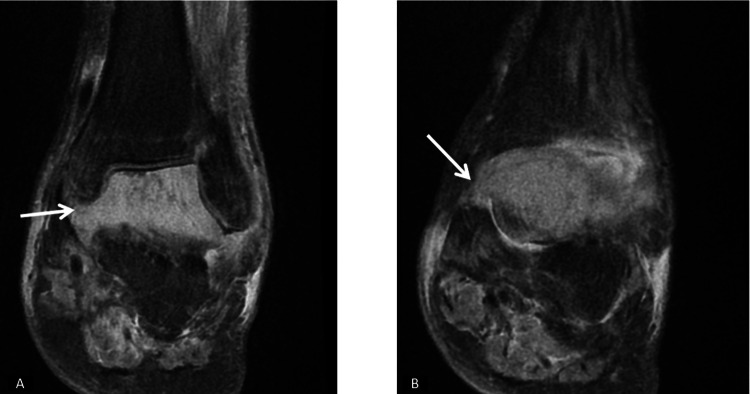
Coronal MRI of the left ankle, obtained with T2-weighted sequences following contrast administration, demonstrates abnormal signal intensity in the ankle with adjacent soft tissue involvement, indicative of potential malignancy. (A) shows involvement around the talus, while (B) highlights involvement in the soft tissue surrounding the talus. The white arrows indicate the area of the lesion. MRI: magnetic resonance imaging

Initially, a contrast-enhanced MRI of the ankle was ordered due to suspicion of osteomyelitis. Following the MRI scans, a heterogeneous contrast-enhancing lesion, measuring 5x5x6 cm, was observed, causing significant destruction in the talus, obliterating the subtalar joint spaces, and causing destruction in the medial malleolus, while also surrounding the tendons of the tibialis posterior and flexor muscles (Figures 2-4).

Subsequently, a PET scan was performed, which strongly suggested a malignancy, and the patient underwent an open biopsy of the lesion. PET imaging revealed a highly hypermetabolic mass lesion in the left ankle, causing extensive destruction of the talus, extending to the medial malleolus of the left tibia, with significant extensions into the soft tissues posteriorly and medially around the ankle, and extending proximally along the leg vessels.

The biopsy material was sent for pathological and microbiological analysis. The patient was discharged from the hospital the following day and scheduled for follow-up appointments in the outpatient clinic. Immunohistochemically, atypical cells were CD20 (+), BCL2 (+), MUM1 (+), CMYC positive in 5-30% of cells, cyclin D1 (-), and CD30 (-). Epstein-Barr virus early ribonucleic acid (RNA) transcript was not detected using the in situ hybridization method (Epstein-Barr encoding region (EBER)-negative). The background showed a small number of mature T lymphocytes staining with CD3. The Ki-67 proliferation index was high (80-90%). These findings were consistent with a diagnosis of high-grade B-cell NHL. Additionally, there was no evidence of bone marrow involvement. 

During the preoperative preparation, no abnormalities were detected in the evaluation of the patient's blood parameters. Following the confirmed diagnosis of NHL, additional detailed blood tests were conducted in the internal medicine department for further assessment (Table 1).

**Table 1:** Preoperative blood test results of the patient, highlighting key hematological, biochemical, and serological parameters used to evaluate the patient's condition prior to biopsy and confirm the absence of systemic abnormalities. WBC: white blood cell; HGB/HCT: hemoglobin/hematocrit; PLT: platelet; ESR: erythrocyte sedimentation rate; CRP: C-reactive protein; LDH: lactate dehydrogenase; AST/ALT: aspartate aminotransferase/alanine aminotransferase; HIV Ab: human immunodeficiency virus antibody; HBsAg: hepatitis B surface antigen

Parameters	Patient's value	Normal range
WBC	10.17	4.3-10.3 10^3^/µL
HGB/HCT	11.5/32.5	13.6-17.2 g/dL/42-52%
PLT	397	156-373 10^3^/µL
ESR	43	0-15 mm
CRP	23.92	0-5 mg/L
LDH	250	<250 U/L
Serum creatinine	0.56	0.7-1.2 mg/dL
AST/ALT	15/20	<40 IU/L
HIV Ab	Negative	Negative
HBsAg	Negative	Negative

After the diagnosis of diffuse large B-cell NHL was confirmed, the standard R-CHOP chemotherapy regimen was administered, consisting of rituximab, cyclophosphamide, doxorubicin, vincristine (Oncovin), and prednisolone. The treatment was given in 21-day cycles for a total duration of six cycles. The patient also received radiotherapy to the affected region, with a total dose of 40 Gy administered as part of the treatment protocol. At the six-month follow-up after surgery, the patient reported a significant reduction in pain. Routine follow-up appointments were maintained. A follow-up PET-CT scan showed no evidence of recurrence or metastasis.

## Discussion

NHL refers to a diverse group of malignancies that arise within the lymphatic system and predominantly affect lymphocytes, a critical component of the immune system. Unlike Hodgkin lymphoma, NHL is distinguished by the absence of Reed-Sternberg cells, which are characteristic of the former. NHL can originate from different types of lymphocytes, such as B cells, T cells, and natural killer cells, leading to various subtypes with unique clinical presentations and treatment pathways. The symptoms of NHL can vary significantly depending on the subtype and the location of the disease, with common signs including lymph node enlargement, fever, night sweats, fatigue, and unexplained weight loss. However, our patient did not exhibit any of these symptoms and presented solely with diffuse pain around the ankle [5]. The diagnostic process often involves a combination of imaging techniques, such as CT and PET scans, along with histopathological evaluation of biopsy samples. Treatment modalities for NHL are individualized and may include chemotherapy, radiation therapy, immunotherapy, targeted therapy, or stem cell transplantation, tailored to the specific subtype, disease stage, and the patient's overall health condition.

Following the diagnosis of NHL through a biopsy procedure in our patient, consultations were requested with the relevant departments to initiate chemotherapy and radiotherapy. Without undertaking any additional surgical interventions, we collaborated with the internal medicine department for the treatment of the primary disease.

Although NHL more commonly presents with the involvement of lymphatic system organs, it can also, albeit rarely, affect the musculoskeletal system. While musculoskeletal involvement more frequently occurs in the pelvic region, it can exceptionally manifest in the foot and ankle joints [6]. What made our patient unique was the exceedingly rare localization of the disease in the ankle as its primary manifestation. Despite initially suspecting osteomyelitis or malignancy, the pathological diagnosis confirmed NHL. Although lymphoproliferative diseases like NHL are rarely encountered in the ankle, they should not be excluded from the differential diagnosis. A thorough examination, including the evaluation of blood parameters, is essential, and a multidisciplinary treatment approach should be considered. Furthermore, coordinated management with the internal medicine department is critical for treating patients with lymphoproliferative diseases.

In a study conducted by Binici and colleagues, it was shown that, albeit very rarely, NHL can originate from the skeletal muscle. However, this is even less common than cases originating from bone and joint and should be considered as part of the differential diagnosis [7].

Studies have also identified the elevation of serum lactate dehydrogenase (LDH) as a prognostic indicator [8]. For our patient, no abnormalities were detected in blood parameters during the preoperative period. Additionally, no elevation in LDH levels was observed during the postoperative period when the patient was referred to the internal medicine department.

Primary bone lymphoma (PBL) generally has a favorable prognosis, with five-year survival rates of 80-95% in localized cases, especially with combined chemotherapy and radiotherapy. Early-stage disease and normal LDH levels are associated with better outcomes.

## Conclusions

While lymphoproliferative diseases primarily involve the lymphatic system, they can, in rare cases, manifest clinically by affecting the joints of the musculoskeletal system. Such atypical presentations pose significant diagnostic challenges, as they can mimic more common conditions like tumors or osteomyelitis. This highlights the importance of maintaining a high index of suspicion for lymphoproliferative diseases, such as NHL, in patients presenting with joint-related symptoms and atypical imaging findings.

A multidisciplinary approach, including thorough imaging studies, histopathological examination, and collaboration between orthopedic surgeons, oncologists, and pathologists, is crucial for accurate diagnosis and effective management. Recognizing rare presentations of diseases like NHL in musculoskeletal sites can prevent misdiagnoses and ensure the timely initiation of appropriate treatment, improving patient outcomes. This case underscores the importance of considering lymphoproliferative diseases in the differential diagnosis of joint involvement and emphasizes the necessity of a holistic and coordinated approach in their management.
